# Impact of Shelf-Life Simulation on a Self-Adhesive Composite: Polymerization Kinetics, Chemical and Color Stability

**DOI:** 10.3290/j.jad.b4368821

**Published:** 2023-09-19

**Authors:** Helena Seoane, Filipa Chasqueira, Ana Mano Azul, Mário Polido, António HS Delgado

**Affiliations:** a Dentist, Egas Moniz School of Health and Science, Monte de Caparica, Almada, Portugal. Performed the experiments, wrote the manuscript.; b Lecturer, Egas Moniz Center for Interdisciplinary Research (CiiEM), Monte de Caparica, Almada, Portugal. Formal analysis, reviewed and edited the manuscript.; c Professor, Egas Moniz School of Health and Science, Monte de Caparica, Almada, Portugal. Resources, supervision and conception, reviewed the manuscript.; d Lecturer, Egas Moniz School of Health and Science, Monte de Caparica, Almada, Portugal; Division of Biomaterials and Tissue Engineering, UCL Eastman Dental Institute, University College London, Hampstead, UK. Conception, statistical analysis, wrote and edited the manuscript.

**Keywords:** color stability, resin composite, polymerization, self-adhesive composite, shelf-life

## Abstract

**Purpose:**

To determine the polymerization kinetics and color stability of a self-adhesive and conventional resin composite after accelerated shelf-life simulation.

**Materials and Methods:**

Two composites were tested – universal Filtek Z250 (3M Oral Care) and self-adhesive Constic (DMG). They were stored for 2 months in an incubator to simulate an Arrhenius aging model (60ºC) and tested at 5 different time points. Polymerization kinetics (n = 3) were studied using an attenuated total reflectance technique (ATR), through continuous FTIR spectral acquisition (20 min). Spectra were obtained before, during and after 20 s of light curing. With the spectral data, qualitative analysis was performed yielding chemical stability, and quantitative data including extrapolated degree of conversion (DC_max_) and polymerization rate (Rp_max_) were assessed. To evaluate color stability (n = 3), a spectrophotometer was used to record CIELAB color parameters. Inferential statistics, including repeated measures two-way ANOVA were carried out at a significance level of 5%.

**Results:**

The composites did not appear to undergo significant chemical changes after 2 months of accelerated aging. There was a significant impact of aging on the mean DC_max_ (p < 0.001). Similarly, a reduction in Rp_max_, measured for both composites, was also noted (ANOVA; Z = 203.7; p < 0.001). The two-way ANOVA confirmed that the composite had no influence on the color stability (F = 0.94; p = 0.34), while aging did (p = 0.013).

**Conclusion:**

Minimal changes in absorbance levels were noted for both composites, without overly affecting their chemical composition. The presence of an acidic monomer did not seem to potentiate the degradation of the self-adhesive composite. This composite even showed greater color stability after aging.

In contemporary dentistry, there is a preference for conservative and ideally biomimetic materials.^55^ As such, modern resin composites (RC) and adhesives are able to mimic most of the natural properties found in teeth.^35^ However, due to their polymeric nature, irreversible changes to their original properties are inevitable, and this limits the longevity of the restoration. Such deterioration of properties over time is referred to as aging.^45^ Thus, the clinical prognosis of RC restorations has a time stamp and remains a particular concern.^26^ Aging mechanisms such as thermal degradation can have a significant impact on the organic matrix of the composite, provoking depolymerization, crosslinking or chain scission events, which also naturally lead to the formation of detrimental by-products.^7^ These alterations affect the polymerization extent of the composite as well as its color stability. It has been proven that inefficient polymerization with a low degree of conversion (DC) of resin-based restoratives translates into poor outcomes. These include a marked reduction in strength of the restorative complex, solubilization of monomers, increased hydrolyzation potential and enzymatic biodegradation.^16,44^ Lower mechanical properties, monomer release, degradation and less color stability are all likely to occur.^1,6^ Indeed, exposure to heat may result in discoloration or fading due to a breakdown of the organic matrix constituents in the polymeric blend. Despite the great development in recent years of composite formulations, the color stability of these materials also remains a major long-term problem, justifying its study.^28,36^

Despite relying on an adhesive protocol that is well established in the literature, with excellent evidence of clinical success, adhesive restorations still have many drawbacks.^12,31,50^ The protocol involves multiple steps, which increases the probability of introducing an error in each one of those steps, it is time consuming, requires ideal conditions and is still very prone to degradation over time.^34,51^ In fact, Van Meerbeek and Frankenberger^49^ have commented that adhesive restorations in suboptimal clinical conditions are not reliable. Owing to the evolution towards streamlining in adhesive dentistry, and in order to make procedures easier, simplification of these materials have been sought.^52^ For these reasons, self-adhesive composites were developed, not only to simplify clinical procedures, but also to eventually eliminate the most sensitive step of the restorative procedure: the correct application of the adhesive.^17,32^ Compared to a conventional composite, self-adhesives are flowable resin-based materials that benefit from the addition of an acidic functional monomer.^22,28^ Some in-vitro and in-vivo studies have shown that these composites, although promising and desirable, still show poor results.^26,30,38^ Their clinical applications are restricted, as they are currently exclusively utilized in small retentive Class I/II cavities, as liners/bases or fissure sealants, and particularly useful in demanding settings such as pediatric dentistry. This highlights that it is imperative to carry out further research to better understandand formulate the next-generation of self-adhesive materials.^14,39^

As mentioned above, the shelf life of composites is a topic of high interest and relevance, since some components of current formulations, namely functional monomers, due to their acidic nature may not be stable and are prone to degradation over time.^12^ As these monomers are a component of self-adhesive composites, and there is a paucity of aging-stability studies on adhesives in general^10,19^ – let alone self-adhesive composites – it becomes pertinent to investigate this topic. This will allow insight into the degradation profile of self-adhesive composites and how they may behave differently from conventional formulations. Furthermore, attenuated total reflectance Fourier-Transform infra-red spectroscopy (ATR-FTIR), in combination with a spectrophotometer allows the rapid study of polymerization kinetics, chemical stability and color of polymeric dental materials, as shown in previous studies.^33,41^

Thus, the main aim of this study was to determine the polymerization kinetics and color stability of a self-adhesive resin composite, comparing it to a conventional resin composite after accelerated shelf-life simulation. This serves to assess its degradation profile in response to simulated aging. The null hypotheses were that, after 2-month simulated shelf-life aging, there were (1) no significant qualitative changes in the ATR-FTIR spectra of the self-adhesive composite after aging; (2) no significant changes in the extrapolated final degree of conversion and maximum rates of polymerization upon material comparison, and (3) no significant changes in the color stability.

## Materials and Methods

### Materials and Sample Size Calculation

The materials used in this study are shown in Table 1.

**Table 1:** Resin composites used in this study (composition, source, batch and instructions provided by the manufacturer)

	Constic	Filtek Z250
Organic matrix	Bis-GMA (15-35 %) TEG-DMA (<45 %) 10-MDP (N/A%)*	UDMA (1-10%) Bis-EMA (1-10%) Bis-GMA (1-10%) TEG-DMA (1-5%)
Filler Type and Size	Barium aluminosilicate (0.02–2.3 µm)	Silica, aluminium oxide and zirconia particles (0.01–3.5 µm)
Filler load (vol %)	43 vol%	60 vol%
Color	A2	A2
Manufacturer	DMG (Hamburg, Germany)	3M Oral Care (St Paul, MN, USA)
Batch	237836	NC86111
Instructions	Place increments with a maximum thickness of 2 mm Light cure for 20 s	Place increments with a maximum thickness of 2.5 mm Light cure for 20 s

Since the appropriate sample size had already been established in a previous study,^16^ there was no need to conduct a priori sample-size calculations for polymerization kinetics data in this study. The sample size for color stability was calculated in G*Power 3.1, for a power of 80% and alpha error probability of 5%, by estimating the effect size from ∆E means, based on the results of a pilot study.

Initially, this study also featured Vertise Flow (Kerr; Orange, CA, USA). However, during simulated aging at 60ºC, pre-polymerization phenomena were observed in Vertise Flow after only 24 h, rendering it unsuitable to generate data. As a result, only Constic and Filtek Z250 were included in the analysis.

### Accelerated Aging Model

To study the shelf-life stability and degradation profile of composites, an accelerated aging protocol was used. This was based on an Arrhenius model, which works in accordance with the 10-degree rule. This states that a sequential increase of 10°C above room temperature is able to double the reaction rate.^4,9^ Therefore, the following equation was used:

$$\mathrm{Real Time}(RT)=\mathrm{Accelerated Aging Time}(AAT)\times Q_{\mathrm{10}}(tAA-tRT/\mathrm{10})$$ (Eq. 1)

where tRT represents the room temperature (22°C), tAA is the accelerated aging temperature (60°C) and Q10 is the reaction rate coefficient (a constant equal to 2). Thus, based on this equation, the formulations were stored for 60 days in an incubator at 60ºC controlled temperature (Memmert INE 400; Schwabach, Germany). This is approximately equivalent to 28 months of storage at room temperature (22°C). The timepoints chosen were 1 day, 1 week, 1 month and 2 months of shelf-life simulation. These timepoints are chosen to capture any immediate or early changes in the material’s properties, other initial changes that may stabilize or progress, and also changes which occur over a somewhat longer period of time.

### Chemical Stability and Polymerization Kinetics (ATR-FTIR)

To determine influence of aging on the chemical composition of the selected composites, samples were taken at each time point (1 day, 1 week, 1 month and 2 months of shelf-life simulation) and an ATR-FTIR spectrum (Spectrum 65, Perkin-Elmer; Waltham, MA, USA) was acquired. Initially, regarding qualitative analysis, a graph was constructed containing the individual spectrum of each time point, which allowed quick comparison of peaks and troughs, also by employing difference spectra, as explained in previously published studies.^15,16^ In addition, ATR-FTIR data were used to assess the polymerization kinetics, before, during, and after light curing for 20 min, using TimeBase software (Perkin-Elmer). Composite disks (2 mm thick, 10 mm internal diameter) were made by dispensing the composite into carbon-steel circlips placed on the ATR diamond. While spectral acquisition was already running, the disks were covered with an acetate sheet and their top surface was individually irradiated for 20 s with an LED blue light curing unit (LCU DB686; COXO Medical Instruments; Guangzhou, China), with a measured peak irradiance of 950 mW/cm^2^ (monitored using an analogic radiometer), contacting the acetate, at zero distance. Light curing began an average of 20 ± 5 s after the start of the spectral acquisition. Maximum extrapolated degrees of conversion (DC_max_) were determined, and rates of polymerization (Rp_max_) were derived from the slope of the conversion-over-time graph, following the method described by Delgado et al.^15^

Maximum extrapolated degree of conversion values were calculated using the following equation:

$$D_{C}=\left(h_{0}-ht\right)/h_{0}$$ (Eq. 2)

where h_0_ and h_t_ were taken as peak absorbance at 1319 cm^-1^ wavenumber, above background at 1336 cm^-1^ initially and at time t, after 1200 s.^16^ Dc_max_ was obtained by linear extrapolation of late time DC values, versus inverse time to zero (as inverse of zero is infinity).

### Color Stability

Color stability was also tested on RC disks (n = 3), using a commercial spectrophotometer Spectroshade Micro (MHT, Niederhasli, Switzerland), evaluating the composites at each time point. Disks of set dimensions (10x1 mm) using metal circlips were made and polymerized in four overlapping polymerization cycles of 20 s each, in direct contact with an acetate sheet. The light-curing unit and its parameters were the same as mentioned above. The color measurement of all samples was performed, recording the three CIELAB color parameters (L*, a* and b*). The sequence was repeated for each time point, in accordance with ISO 7491:2000.^20^

To verify the existence of alteration in color, a colorimetric analysis was performed by calculating delta E (∆E) with the following equation:

$$\Delta E_{Lab}=\sqrt[{0}]{{\left(\Delta L^{*}\right)}^{2}+{\left(\Delta a^{*}\right)}^{2}+{\left(\Delta b^{*}\right)}^{2}}$$ (Eq. 3)

where ∆E_Lab_ represents the difference between the final measurement and the initial measurement of the color parameter “L”; the same concept applies to ∆a and ∆b, as these are the difference between the final and the initial measurements of the color parameters “a” and “b”, respectively. The L*, a*, b* values present in equation 3 are necessary for determinomg the value of ∆E, and were collected through spectrophometric measurements. In this study, the ∆E values were obtained considering T_0_ as the initial color measurements, for all time points.

### Statistical Analysis

SPSS v 26.0 for Mac (IBM; Armonk, NY, USA) was used for hypothesis testing. After confirming normality assumptions using the Shapiro-Wilk test, a two-way repeated-measures ANOVA was performed to compare the Dc_max_ and Rp_max_ means obtained for the two composites, using a Bonferroni post-hoc test for multiple comparisons. The two independent variables were: (1) time (independent time points) and (2) the type of RC (Filtek Z250/Constic). The level of significance was set at 5%. A two-way ANOVA, using the same post-hoc test, was employed to test the color stability.

## Results

### Chemical Stability

**Fig 1:**
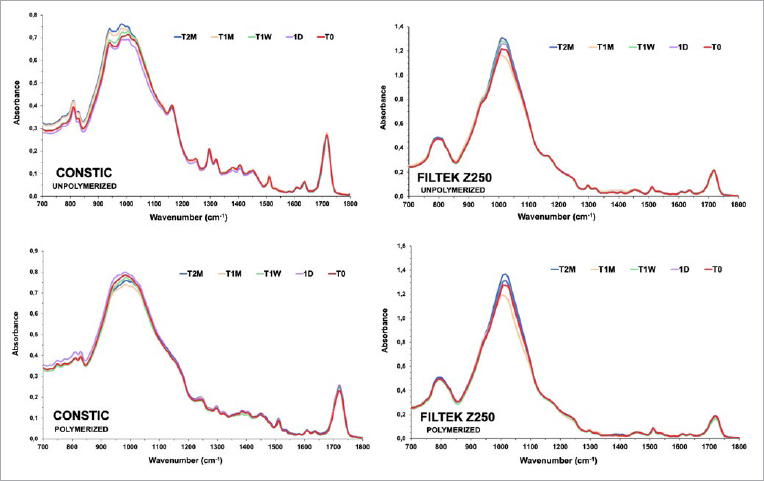
Unpolymerized and polymerized ATR-FTIR spectra for each timepoint (T_0_ to T_2_M), showing changes in the absorbance levels (peaks and troughs) for both composites. T_0_: initial time; T_1_D: after 24 h; T_1_W: after 1 week; T_1_M: after 1 month; T_2_M : after two months.

ATR-FTIR peak changes for both composites can be seen in Fig 1. It is possible to observe slight differences in the absorbance of the composites after shelf-life simulation. For Filtek Z250, a slight increase in the absorbance between the 1000 cm^-1^ region can be seen, while the polymerized spectra for Constic shows an increase in the 700 – 1050 cm^-1^ region.

Comparing the unpolymerized to the polymerized spectra of Filtek Z250, the following changes were noted: the stretching vibrations of [v(C=C)] at 1610 cm^-1^ and [v(C=O)], corresponding to bis-GMA, decreased. The typical absorbance levels N-H vibrations (compatible with UDMA) at 1140 cm^-1^ and 1530 cm^-1^ decreased. Finally, absorbance levels derived from the [v(C-O-C)] of methacrylates at 1120 cm^-1^ decreased.

Similarly, with Constic, the following functional groups showed slight changes: the stretching vibrations of [v(C=C)] at 1610 cm^-1^ and [v(C=O)], corresponding to bis-GMA, decreased; absorbance levels derived from the [v(C-O-C)] of methacrylates at 1120 cm^-1^ decreased. and the stretching vibrations of [v(P-O)] and [v(P-O-C), between 950 cm^-1^ and 1250 cm^-1^ showed an increase, corresponding to changes in 10-MDP. Most of these changes are compatible with methacrylate co-polymerization. Qualitatively, none of the commercial composites seemed to undergo significant changes in their chemical composition after the chosen period of shelf-life simulation at 60ºC.

### Polymerization Kinetics

**Fig 2:**
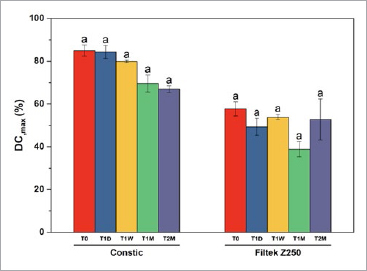
Bar chart showing degree of conversion means, while error bars represent standard errors. Within composites, values did not differ significantly (Bonferroni, p>0.05).

With aging, it was possible to note an overall reduction in the degree of conversion in both composites. The overall impact of aging was further confirmed with the ANOVA model, with sphericity assumed (repeated measures ANOVA; Z = 4,787, p = 0.01), although differences were not statistically significant within the same composite. On average, Constic showed higher DC_max_ mean results compared to Filtek Z250. Thus, the type of RC had a statistically significant impact on the DC_max_ mean (p < 0.001). However, as mentioned above, when considering the multiple comparisons, no statistically significant differences were found. The mean results are shown in Fig 2.

**Fig 3:**
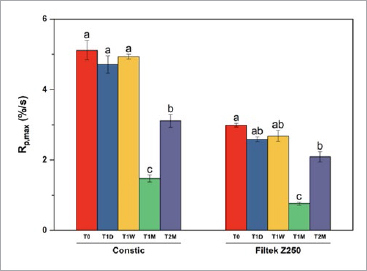
Bar chart representing the Rp_max_ means with standard error of the mean as the error bars. Bars showing different letters within the same composite are significantly different (Bonferroni, p < 0.05). Constic shows differences from T_0_, T_1_D and T_1_W to T_1_M and T_2_M. Filtek Z250 exhibits changes from T_0_ to T_1_M and T_2_M, and also from T_1_D and T_1_W to T1M.

Similarly, with time, a reduction in Rp_max_ means were also registered for both composites (repeated measures ANOVA; Z = 203,722; p < 0.001). However, the greatest reduction for both materials was seen after shelf-life simulation for 1 month. The impact of varying the type of composite also gave different mean results, with Constic showing the same trend as DC_max_, with higher values compared to Filtek Z250. The bar chart with multiple comparisons is shown in Fig 3.

In order to organize all the results obtained, the descriptive statistics were included in a single table (Table 2). In this table, it is possible to observe a decreasing trend, with time, for both parameters DC_max_ and Rp_max_, in the two materials that were tested. On average, it is also possible to see that Constic outperformed Filtek Z250 in both parameters.

**Table 2:** Values of descriptive statistics for DC_max_ and Rp_max_ of Constic and Filtek Z250 in each timepoint, means and standard error (in parentheses)

RC Time	DC_max_		Rp_max_	
	Constic	Filtek Z250	Constic	Filtek Z250
T_0_	85.0 (4.5)^Aa^	57.7 (5.7)^Ba^	5.1 (0.5)^Aa^	3.0 (0.1)^Ba^
T_1_D	84.3 (5.3)^Aa^	49.4 (6.9)^Ba^	4.7 (0.4)^Aa^	2.6 (0.1)^Bab^
T_1_W	79.9 (0.9)^Aa^	53.9 (2.2)^Ba^	4.9 (0.1)^Aa^	2.7 (0.3)^Bab^
T_1_M	69.6 (6.9)^Aa^	38.9 (6.1)^Ba^	1.5 (0.2)^Ac^	0.8 (0.1)^Bc^
T_2_M	67.0 (2.7)^Aa^	52.8 (16.6)^Ba^	3.1 (0.3)^Ab^	2.1 (0.3)^Bb^

T_0_: initial time; T_1_D: after 24 h; T_1_W: after 1 week; T_1_M: after 1 month; T_2_M : after two months. Different superscript lowercase letters indicate statistically significant differences in columns (Bonferroni, p < 0.05). Different superscript uppercase letters indicate statistically significant differences in rows, comparing the different composites. For DC_max_ and Rp_max_, 99% statistical power was achieved (post-hoc power analysis using G* Power v3.1.9).

### Color Stability

**Fig 4:**
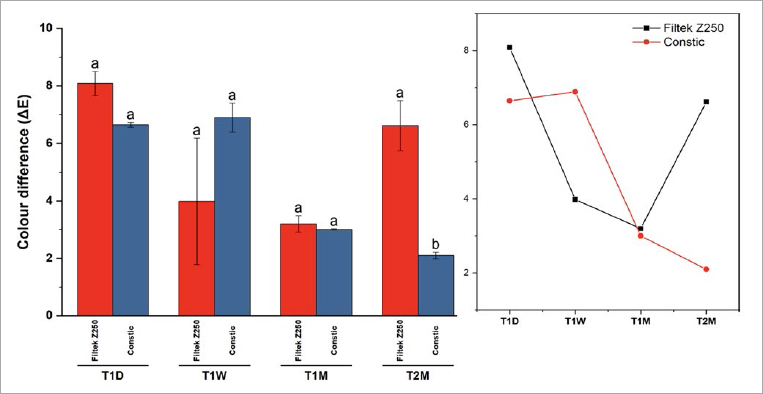
Bar chart representing the color difference means, with standard error of the mean as the error bars. Bars showing different letters within the same composite are significantly different to each other (Bonferroni, p < 0.05). A significant difference was noted at the final timepoint (2 months), when Constic showed less color variation than Filtek Z250 (Bonferroni, p < 0.001). Trend lines are shown on the right. Regarding color stability, 87% statistical power was achieved (post-hoc power analysis using G* Power v3.1.9).

Regarding color stability, two-way ANOVA confirmed that the type of composite did not have an overall influence on the color stability (F=0.94; p = 0.34), while the aging factor did (two-way ANOVA, F=4.6; p = 0.013). Figure 4 illustrates the color variation within each timepoint for the different composites. A similar trend was seen except in the final measurement, in which Constic showed significantly less color variation when compared to Filtek Z250 (Bonferroni, p < 0.001).

## Discussion

The present investigation determined the impact of shelf-life simulation on the properties of a commercial self-adhesive composite, i.e., polymerization kinetics, chemical and color stability, by comparing it to a widely used universal restorative composite. Functional monomers included in contemporary self-etch or universal adhesives, self-adhesive cements and flowable self-adhesive composites have acidic moieties, which generally comprise carboxylic acid, phosphate or phosphonate groups.^48^ As reported by Shibuya et al,^42^ these acidic monomers have a higher tendency to dissociate and degrade. They are likely to lend long-term instability to the chemical composition of adhesives.^42^ Similarly, Ma et al^24^ also describe the impact of storage temperature on the degradation of adhesives. It is expected that degradation in adhesive formulations would be quite different in viscous, non-solvated and hydrophobic composites. However, to the best of our knowledge, no stability study has been published on self-adhesive composites that disclosed the role of functional monomers in their degradation potential.

Shelf-life simulation and accelerated aging studies are common and very useful when testing the stability of polymeric materials.^12^ Composites undergo changes in their physical properties from the date of manufacture, which influences their clinical prognosis. In this study, a simples Arrhenius model was used, based on the 10-degree (ºC) rule. Polymer degradation is evaluated by the deterioration of their physico-chemical properties. Depending on the temperature to which they are submitted, the rate of deterioration of these properties varies. With aging under normal shelf-life conditions, it may take a long time to verify aging-dependent changes. Accelerated aging protocols expose these materials to high temperatures, increasing chemical reaction rates. This in turn facilitates chemical processes such as oxidation, chain scissions or the formation of new cross-links, all of which may be thermally driven.^56^

Although the absorbance levels shown by ATR-FTIR spectra of both composites were slightly higher after aging, in the unpolymerized and polymerized states, the these values did not appear to be qualitatively different after accelerated aging at 60ºC. These results are consistent with previous reports.^2,16^ Thus, the results do not allow rejection of the first null hypothesis. In Constic, a slight increase was detected in the absorbance of all methacrylate monomers in the region of 950 – 1250 cm^-1^. In monomers such as 10-MDP, the phosphate group (P-O and P-O-C) produces a broad peak at the 1000 cm^-1^ region.^16^ The presence of acidic monomers in self-adhesive composites could make their ester linkages more susceptible to degradation over time. However, in this study, the 10-MDP present in Constic did not seem to accelerate its degradation, since the chemical degradation profile was very similar in the two materials tested. Self-adhesive composites do not contain any water or solvents, which means that thermal degradation will not provoke hydrolysis phenomena. For both composites, however, the filler absorbance region showed higher values with aging. This may be related to thermal degradation of the silane coating of the filler particles,^3^ which consequently would expose the fillers. When this happens, they can be contacted by the ATR diamond, giving higher absorbance readings since they are strong oscillators. Chemical-thermal instability of filler-silane coupling has been described before and may also result in cluster agglomeration.^11^

Polymerization parameters are important to characterize and predict the behavior of composites, since they will directly relate to the final properties of the polymeric blend, such as their mechanical, biological, optical, and adhesive behavior.^14,36^ The null hypotheses for DC_max_ and Rp_max_ were both accepted, since the trends seen in the self-adhesive composite with aging were similar to the ones seen in the conventional Filtek Z250, although both parameters were higher with Constic. These differences occur mainly due to their different viscosities. Resin-based materials with lower filler loads, such as self-adhesive flowable composites, inevitably have reduced initial viscosity.^5^ This increases monomer mobility, facilitating active chain collisions,^16^ which would explain the higher DC_max_ as well as the higher rates seen in this study. The values measured for Constic at T_0_ are very consistent with previous DC_max_ values recorded for this composite.^14^ As for Filtek Z250, D’Alpino et al^12^ demonstrated good stability with accelerated aging at 37ºC in terms of polymerization kinetics, which corroborates the findings of the present study. For DC_max_, a decreasing trend was seen with aging. Aging effects may translate into a premature polymerization reaction and consumption of inhibitors within the unset mixtures.^22^ Also, discoloration from aging may reduce effective light penetration.

The changes seen in Rp_max_ manifested as faster or slower rates, depending on the timepoint studied. Since light intensity was fixed and standardized, these differences are likely due to changes in the photoinitiator chemistry and viscosity of the initial mixtures.^24,43^ Polymerization rates vary with temperature; even modest increases of temperature affect curing rates. This has been verified in short-term storage of composites at temperatures similar to the 60ºC used in this study.^47^ However, long-term storage induces chemical changes in the organic matrix of the mixtures, which are also responsible for a decrease in mechanical properties, owing to inefficient polymerization.^8^ The initial decrease in the polymerization rate may be due to the formation of cross-links between the resin molecules, which can reduce the mobility of the monomers and retard the reaction. As the resin continues to age, the crosslinks may break down and the monomers may become more mobile, leading to an increase in rates.^18^ The same applies to the formation of intermediate products or depletion of reactants, responsible for decelerating the reaction.^53^ With further aging, these may be consume or cleared, sparking a subsequent increase in rates. This trend was verified for both composites tested.

The third null hypothesis was rejected, since significant differences in the color stability of the self-adhesive vs conventional composite were observed after 2 months of shelf-life simulation. Some authors have reported that artificial aging affects color stability due to the chemical changes in the composite’s components and to its surface microstructure, such as oxidative chain cleavage and changes to the crystallinity. Crystallinity and inorganic fillers are known to act as barriers to monomer mobility. They can also affect light dispersion through the material. The perception of color is related to the light reflection, which is known to be affected by intrinsic changes in the organic matrix, amines, and initiators present in RC and as a result of aging.^54^ Regarding the filler particles, smaller filler particles or higher filler contents lead to greater light scattering. Of the composites tested here, Filtek Z250 has a greater filler content than Constic; the former also revealed less color stability after 2 months of aging.

RCs normally retain a yellow color even after being light cured, since camphorquinone, the commonly used photoinitiator, is a yellowish particle that has not been completely consumed. In fact, there is a correlation between a more yellow color of the material and a higher percentage of unreacted molecules.^29^ In the present study, previous activation of camphorquinone by heat (60ºC) was almost certain, so the final balance of its activation rate was high, which justifies the increase in the L* parameter at all timepoints. The color change compared to T_0_ became less significant over time, which is expected due to the lower availability of the photo-initiation system at the moment of light curing.^25,40^ However, it showed a subsequent increase in color change after 2 months, probably due to degradation of the organic matrix, with subsequent exposure of filler particles. A more evident effect likely lies in the difference of filler loads between the two composites.

To the best of our knowledge, studies evaluating polymerization kinetics and color stability of dental composites after long-term storage at high temperature are rare. Results featuring polymerization kinetics after short-term heating have been previously reported,^13^ but limited data exists on the impact of the chemical changes after accelerated aging. The speculation that a functional acidic monomer such as 10-MDP could affect the degradation profile of the self-adhesive composite was not proven in this study, since the aging trends were very similar for both materials. The deleterious effect of temperature on the chemical changes is more relevant in the context of storage of adhesives. Storage conditions of solvated mixtures, which often contain water (as is the case in adhesives), tend to facilitate hydrolysis phenomena in functional acidic monomers.^46^ This does not occur in viscous monomer mixtures. In fact, regarding color stability, the flowable self-adhesive composite even showed better results after the total aging period (2 months). Moreover, this study provides a simple, yet very effective aging model that can be used to screen important properties in dental polymeric materials after potential organic matrix degradation.

It is important to address the limitations of the present study. Under the conditions employed here, the Arrhenius aging model used does not account for factors such as moisture, exposure to pH variations, acid challenges and mechanical loading, which are also known to affect the performance of RCs over time. Furthermore, Arrhenius models only provide a rough estimate of the material’s long-term performance, and the predicted performance may not always match the actual behavior of the material in real-life applications. As such, the results of accelerated aging tests should always be interpreted with caution and in the context of other factors that may affect the material’s performance. The accelerated aging protocol that equalled 27 months of shelf-life simulation continued 10 months beyond the 17-month shelf-life claimed by the manufacturer for Constic. Even with this extended aging period, the polymerization kinetics and color stability of Constic were not significantly affected. Furthermore, the performance of Constic was comparable to that of Filtek Z250, an RC with a longer shelf life: 36 months. However, the limitations of our study prevent us from drawing any definitive conclusions regarding the reliability of Constic for clinical use beyond its stated shelf-life. The accelerated aging protocol used does not fully replicate the conditions that the material would encounter over time.

## Conclusion

The two RCs tested showed good stability upon accelerated aging at 60ºC, with acceptable polymerization parameters and minimal changes in their ATR-FTIR chemical spectra. Regarding color stability, after 2 months of shelf-life simulation through accelerated aging, the self-adhesive flowable composite Constic outperformed Filtek Z250. The present findings did not provide evidence to suggest that the presence of an acidic functional monomer in Constic had a significant impact on its degradation profile for the time period studied.

## Clinical relevance:

The self-adhesive composite tested showed a degradation profile similar to that of the universal resin composite. The addition of an acidic functional monomer to its chemical composition did not promote its degradation in a shelf-life simulation model.
